# Complete Genome Sequence of *Halomonas* sp. Strain FeN2, a Novel Cathode-Oxidizing Bacterium Isolated from Catalina Harbor Sediments

**DOI:** 10.1128/MRA.00862-21

**Published:** 2021-11-18

**Authors:** Linda Vu, Joshua D. Sackett, Edmund Leach, Elizabeth Wilbanks, Annette R. Rowe

**Affiliations:** a Department of Biological Sciences, University of Cincinnati, Cincinnati, Ohio, USA; b Department of Ecology, Evolution, and Marine Biology, University of California, Santa Barbara, Santa Barbara, California, USA; University of Southern California

## Abstract

We report the complete, closed, circular genome of *Halomonas* sp. strain FeN2, a metabolically versatile electrotroph that was isolated from Catalina Harbor sediments. The 4.8-Mb genome contains 4,286 protein-coding genes and has complete glycolytic, tricarboxylic acid, glyoxylate, pentose phosphate, and reductive pentose phosphate pathways. FeN2 also contains genes for aerobic and anaerobic (denitrification) respiration.

## ANNOUNCEMENT

Lithotrophic microorganisms play an important role in biogeochemical cycling in marine sediment ecosystems (1–4). However, their metabolisms remain poorly characterized, especially those that use solid-phase minerals (5–8). Extracellular electron transfer (EET), the process by which microorganisms transfer electrons to and from solid-phase surfaces, such as minerals and electrodes, has been extensively studied in only a few model organisms that predominantly perform mineral reduction, including Shewanella oneidensis and *Geobacter* strains (9). The prevalence and diversity of EET mechanisms involved in mineral oxidation are largely unknown. Here, we present the complete genome sequence of *Halomonas* sp. strain FeN2, a new *Halomonas* strain isolated for its ability to perform oxidative EET.

*Halomonas* sp. strain FeN2 was isolated from Catalina Harbor, California, sediment enrichments (1). FeN2 was cultivated aerobically at 30°C and 200 rpm in LB supplemented with 175 mM NaCl, 15 mM MgCl_2_·6H_2_O, and 1 mM CaCl_2_·2H_2_O. DNA for long-read sequencing was extracted using the DNeasy blood and tissue kit (Qiagen, Germantown, MD). Samples were barcoded (native barcoding kit 1D; Oxford Nanopore Technologies, Oxford, UK) and prepared for sequencing (ligation sequencing kit 1D). The library was sequenced using a SpotON flow cell Mk 1 (FLO-MIN106R9). Resulting sequences were base called with Guppy v. 3.0.6 implemented in MinKNOW v. 2.0. Sequence statistics for Nanopore reads were calculated with NanoStat v. 1.5.0 (10). Nanopore long-read sequences were assembled with Flye v. 2.7 (11). For Illumina sequencing, genomic DNA was isolated using the DNeasy PowerSoil kit (Qiagen). DNA libraries were prepared for sequencing using the Nextera XT sample preparation kit (FC-131-1096; Illumina) and submitted for 2 × 250-bp paired-end sequencing using the Illumina HiSeq 4000 platform at the University of California, Davis, DNA Technologies Core Facility. Illumina reads were aligned to the assembly using the Burrows-Wheeler Aligner v. 0.7.17-r1198 (12) and used to polish the long-read assembly using Pilon v. 1.23 (13) with the parameter –fix all. Three rounds of polishing were conducted. Genome statistics and assembly quality were determined with QUAST v. 4.4 (14) and CheckM v. 1.0.18 (15). The genome was annotated using the NCBI Prokaryotic Genome Annotation Pipeline (PGAP). Potential metabolic pathways were identified using the KEGG BlastKOALA functional characterization tool (16) (https://www.kegg.jp/blastkoala). Additional proteins involved in sulfur metabolism were identified by BLASTp v. 2.10.1 (17) as described (18). The nearest neighboring genome was identified with GTDB-Tk v. 1 (19). Default parameters were used for all software unless otherwise specified.

The FeN2 genome is approximately 4.8 Mb, with a G+C content of 55.2% (Table 1). GTDB-Tk identified a draft-quality metagenome-assembled genome of an uncharacterized and uncultured *Halomonas* strain (GenBank accession number GCA_002715145.1) (average nucleotide identity [ANI] of 99.6% and alignment coverage of 65.8%) (20) as the nearest neighbor. BlastKOALA identified complete sets of genes for the Embden-Meyerhof, Entner-Doudoroff, tricarboxylic acid, glyoxylate, pentose phosphate, and reductive pentose phosphate pathways. Pyruvate decarboxylase (*pdc*) was absent, but fermentation genes aldehyde dehydrogenases (*aldB* and *aldH*) and alcohol dehydrogenase (*adh*) were annotated. The genome encodes succinate dehydrogenase, NADH:quinone oxidoreductase, Na^+^-translocating NADH:quinone reductase, terminal cytochrome oxidases *o*, *bd*, and *cbb3*-type, and a bacterial F-type ATPase. All genes needed for dissimilatory nitrate reduction to ammonia are present (*narGHI* and *nirBD*). All genes required for denitrification are present, except for nitric oxide (NO)-forming nitrite reductase. BlastKOALA identified a single gene involved in sulfur metabolism, i.e., thiosulfate dehydrogenase *doxD*, but *doxA* was not identified. Additional putative sulfur metabolism genes found include *fccA* (Q06529), *soxX* (O66187), *dsrC* (D3RSN6), *soeB* (ADC63402.1), *hdrB* (ADJ22501.1), and several sulfurtransferases, including *tusB*, *tusC*, and *tusD*. Our analysis of the FeN2 genome provides a valuable reference to support ongoing bioelectrochemical studies to identify and characterize the genetic basis of oxidative EET and the ecological implications of this process in marine sediments.

**TABLE 1:** Sequencing statistics

Parameter	Finding
BioProject accession no.	PRJNA726530
BioSample accession no. for raw sequence reads	SAMN18934492
GenBank accession no. for genome assembly	CP074200.1
No. of raw paired-end Illumina reads	1,031,497
Estimated Illumina genome coverage (×)	94
No. of raw Nanopore MinION reads (total length [bp])	44,702 (308,894,292)
Nanopore MinION read length (mean [range]) (bp)^*a*^	6,910 (219–93,353)
Nanopore MinION read *N*_50_ (bp)^*a*^	13,681
Estimated Nanopore MinION genome coverage (×)	37
Assembly size (bp)	4,793,405
G+C content (%)	55.2
Estimated genome completeness (%)^*b*^	100
Estimated contamination (%)^*b*^	0.65
No. of contigs	1
No. of protein-coding genes	4,286
No. of tRNA genes	60
No. of rRNA operons	6

a Determined with NanoStat v. 1.5.0 (10).

b Determined with CheckM v. 1.0.18 (15).

### Data availability.

This study is registered under BioProject accession number PRJNA726530. The raw Illumina and Nanopore sequencing reads for *Halomonas* sp. strain FeN2 have been deposited in the Sequence Read Archive (SRA) and are available under BioSample accession number SAMN18934492. The complete genome sequence, with annotations, has been deposited in GenBank under accession number CP074200.1.
